# Supplementary material for: A Capillary-Force-Driven, Single-Cell Transfer Method for Studying Rare Cells
Source: Bioengineering (Basel). 2024 May 24;11(6):542. doi: 10.3390/bioengineering11060542 (PMC11200440; doi:10.3390/bioengineering11060542)
Supplement: Supplementary file 1 [file bioengineering-11-00542-s001.zip › bioengineering-2919520-supplementary.pdf]

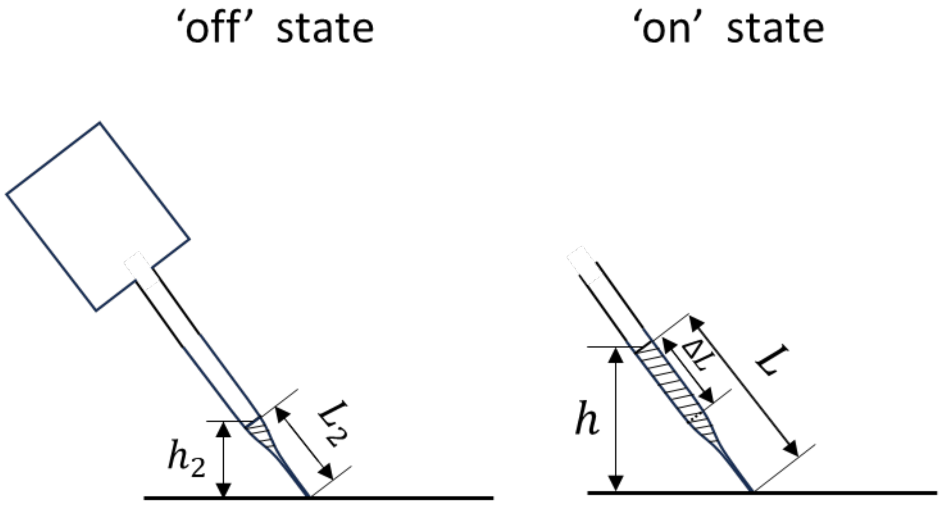

Figure S1. Illustration of the 'off' state and 'on' state of the SCT system.

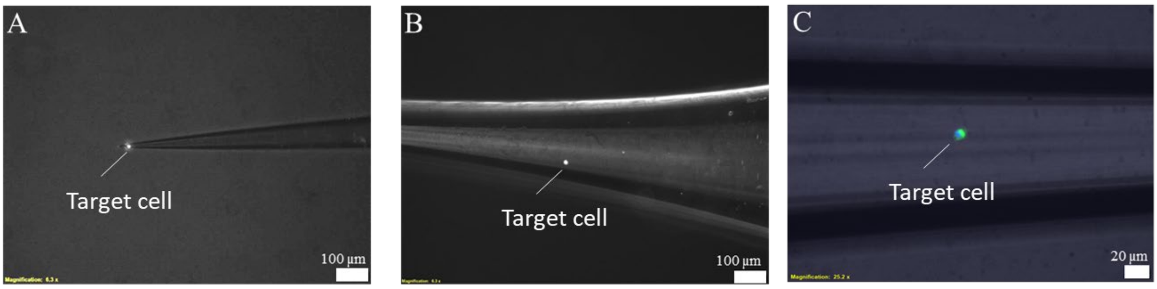

Figure S2. Sample transferred by SCT. (**A**) Aspiration of a single cell to the capillary tip of the SCT system. (**B**) Cell captured in SCT. (**C**) Fluorescently labeled targeted cell in the SCT capillary.
